# Refined Deformable-DETR for Electric Pylon Detection Based on Optical Satellite Image

**DOI:** 10.3390/s26113467

**Published:** 2026-05-31

**Authors:** Jun Yang, Yu Sun, Yingjun Zhao, Donghua Lu, Yuxi Hao, Xianglin Liu

**Affiliations:** 1 Beijing Research Institute of Uranium Geology, Beijing 100029, China; yangjun@briug.cn (J.Y.); zhaoyingjun@briug.cn (Y.Z.); ludonghua@briug.cn (D.L.); haoyuxi@briug.cn (Y.H.); liuxianglin@briug.cn (X.L.); 2 National Key Laboratory of Uranium Resources Exploration-Mining and Nuclear Remote Sensing, Beijing 100029, China

**Keywords:** refined deformable-DETR, electric pylon detection, optical satellite image, query modulation

## Abstract

Automatic detection of electric pylons in optical remote sensing imagery is important for large-scale powerline monitoring, but remains challenging due to complex backgrounds, small target appearances, and large variations in pylon-shadow structures. This paper proposes a Refined Deformable-DETR framework with a Spatial Context-aware Query Modulation (SCQM) module to enhance object query representations. SCQM aggregates image-level contextual information from encoder memory and generates channel-wise modulation vectors to recalibrate object queries before deformable cross-attention, thereby providing image-conditioned channel priors for subsequent query-feature interaction. Experiments on the self-constructed Electric Pylon Remote Sensing Dataset (EPRD) show that the proposed method improves AP from 72.7% to 74.1% and APs from 47.2% to 50.9% compared with the baseline Deformable-DETR. Evaluations on the public Electric Pylon Dataset (EPD) further demonstrate its generalization capability. These results indicate that context-aware query modulation is effective for Transformer-based electric pylon detection in complex remote sensing scenarios.

## 1. Introduction

Electric pylons are critical components of modern power infrastructure, and their operational status is directly related to the stability and safety of power transmission systems. Traditional manual inspection is labor-intensive and inefficient, while unmanned aerial vehicle (UAV)-based inspection is limited in large-scale and high-frequency monitoring scenarios. In contrast, optical satellite remote sensing provides a promising solution due to its wide coverage, high temporal efficiency, and ability to support large-area observations. With the rapid development of high-resolution optical satellite imagery, automatic detection of electric pylons from satellite images has become an essential approach for ensuring power system reliability. Beyond optical imagery, the extraction of powerlines and pylons from remote sensing data has also been widely investigated for infrastructure inventory and power corridor management [1].

In recent years, a growing number of studies have explored the use of optical remote sensing imagery for electric pylon detection. For instance, Tian et al. [2] introduced an enhanced rotation-based detection framework to improve detection accuracy in high-resolution imagery. Chi et al. [3] proposed an EP-YOLOv8 model for efficient pylon detection, demonstrating the feasibility of deep learning-based approaches for this task. These studies have shown promising results; however, achieving robust and accurate detection in complex remote sensing environments remains challenging.

Specifically, electric pylons are often distributed across diverse backgrounds such as farmland, mountains, and forests, making them susceptible to background interference. In addition, variations in imaging viewpoints and altitudes can significantly compress structural features of pylons, leading to substantial appearance variations. To address this issue, some studies have incorporated shadow information as an auxiliary cue. For example, Huang et al. [4] jointly modeled pylons and their shadows to enhance detection performance. Nevertheless, shadow features exhibit significant scale variations under different imaging conditions, which poses additional challenges for stable feature representation.

Recent advances in deep learning, particularly Transformer-based object detection methods, have provided new perspectives for addressing these challenges [5]. The Transformer architecture [6] enables effective modeling of long-range dependencies through self-attention mechanisms. Detection Transformer (DETR) [7] first introduced Transformer into object detection, achieving end-to-end detection via set prediction. Deformable-DETR [8] further improves efficiency and multi-scale modeling by introducing deformable attention. However, when applied to electric pylon detection, Deformable-DETR still exhibits limitations. Small objects in remote sensing images are often characterized by limited pixel coverage, weak appearance cues, and strong background interference, making robust detection particularly challenging [9]. In particular, its object queries lack explicit awareness of global spatial context, which restricts their ability to represent small or context-dependent targets in complex scenes.

In Transformer-based detectors, object queries serve as key representations that directly influence detection performance. Therefore, explicitly modeling and optimizing query representations is crucial for improving detection accuracy, especially in scenarios with large-scale variations. Existing studies such as DINO [10] have shown that improving query initialization can accelerate convergence and enhance performance. However, most approaches focus on query initialization or positional encoding, while the dynamic adaptation of query representations based on image content remains underexplored.

To address these limitations, this paper proposes a Refined Deformable-DETR framework for electric pylon detection. The core idea is to enhance query representation by incorporating global contextual information. Specifically, a Spatial Context-aware Query Modulation (SCQM) module is designed to aggregate global semantic information from the encoder and generate channel-wise modulation vectors. These vectors are then used to adaptively recalibrate object queries before each decoder layer. By dynamically adjusting query representations according to the input image, the proposed method improves the model’s ability to capture multi-scale pylon and shadow features while suppressing background noise. The main contributions of this work are summarized as follows:

1. A Refined Deformable-DETR framework is proposed for electric pylon detection in optical remote sensing imagery, which enhances detection performance by refining object query representations.
2. A Spatial Context-aware Query Modulation (SCQM) module is introduced to enable effective interaction between global contextual information and object queries, improving robustness against scale variation and background interference.
3. Extensive experiments on the self-constructed EPRD demonstrate that the proposed method improves the overall AP by 1.4% over the baseline, with a notable gain of 3.7% for small objects. Additional evaluations on the public EPD further verify the generalization capability of the proposed approach.

The remainder of this paper is organized as follows. Section 2 reviews related work. Section 3 presents the proposed Refined Deformable-DETR framework and the SCQM module. Section 4 reports the experimental settings and results. Section 5 discusses the effectiveness and limitations of the proposed method. Section 6 concludes the paper and outlines future research directions.

## 2. Related Work

In recent years, an increasing number of studies have explored the automatic detection of electric pylons from remote sensing imagery [11,12]. Existing mainstream approaches are predominantly based on convolutional neural networks (CNNs), which improve detection performance by constructing multi-scale feature representations or optimizing detection frameworks. For instance, representative detectors such as Faster R-CNN and YOLOv3 have been widely applied to transmission tower detection tasks, demonstrating the effectiveness of deep learning methods in this domain [13,14,15]. Building upon these approaches, researchers have further introduced multi-scale feature enhancement strategies to address challenges such as large-scale variations and complex backgrounds in remote sensing images. For example, NAS-FPN [16] learns a scalable feature pyramid architecture to enhance multi-scale feature representation, thereby improving detection performance across objects of varying sizes.

However, electric pylons typically exhibit slender structures and are easily disturbed by complex backgrounds in high-resolution remote sensing imagery. Their associated shadows can provide useful contextual cues for detection, and Huang et al. [17] improved slender-object detection by jointly modeling electric pylons and their shadows. Nevertheless, shadow appearance varies substantially with imaging viewpoint and illumination conditions. Such variations make it difficult for convolution-based local feature extraction methods to establish stable associations between pylons and shadows across different scenes, thereby limiting model generalization.

To tackle the common challenges of scale variation and background interference in remote sensing images, previous studies have mainly focused on feature enhancement and loss function optimization. In broader detection-related tasks, robust feature interaction and complementary information modeling have also been explored to improve detection robustness under complex imaging conditions, such as RGB-T salient object detection with modality-specific and modality-complementary feature modeling [18]. At the feature level, Feature Pyramid Network (FPN) [19] and its variants, such as PANet [20] and BiFPN [21], enrich feature representations through cross-scale path aggregation. SCRDet [22] further integrates multi-scale features with attention mechanisms to suppress background clutter and enhance target responses. At the loss level, IoU-balanced Loss [23] improves training by adjusting gradient contributions based on IoU values, while Equalization Loss [24] mitigates class imbalance through gradient reweighting. Although these methods improve multi-scale perception to some extent, they fundamentally rely on progressively stacked local receptive fields, making them inadequate for modeling structured targets like “tower–shadow” pairs, which involve long-range dependencies and drastic scale variations.

In contrast, Transformer-based architectures can directly model global dependencies through self-attention mechanisms, showing strong potential in complex background modeling. Recently, Transformer-based object detection methods have advanced rapidly, and recent reviews have identified query design, attention refinement, and convergence acceleration as important directions for improving DETR-based detectors [25]. For example, Conditional DETR [26] introduces spatial conditional information to optimize cross-attention, DAB-DETR [27] represents queries as dynamic anchor boxes to enhance expressiveness, and DN-DETR [28] improves training stability and detection accuracy through denoising strategies. In addition, Anchor DETR [29] and SAM-DETR [30] further enhance detection performance from the perspective of query design. However, most existing approaches focus on query initialization or positional priors, while overlooking the dynamic quality optimization of object queries during the decoding process. In transmission tower detection scenarios, static or generic query embeddings struggle to adaptively capture shadow scale variations caused by viewpoint changes, thereby limiting feature representation capability under complex conditions.

Meanwhile, channel-wise feature recalibration mechanisms have been proven effective in enhancing feature discriminability. Methods such as SENet, CBAM, and ECA-Net introduce global context to dynamically reweight feature channels, achieving significant improvements with minimal computational overhead [31,32,33]. Furthermore, approaches like Dynamic Head [34] and CondConv [35] incorporate input-conditioned dynamic modulation, enabling adaptive modeling of scale and semantic information. These studies suggest that context-aware dynamic modulation is a promising strategy for handling complex scale variations.

Motivated by these observations, we propose a Spatial Context-aware Query Modulation (SCQM) module to enhance object query representations in the decoder. Unlike conventional methods that recalibrate feature maps, SCQM uses global contextual information from the encoder to generate dynamic channel-wise modulation vectors for the query representations entering each decoder layer. This design enables object queries to adapt to image-specific context and improves robustness against background interference and shadow scale variations.

## 3. Method

### 3.1. Deformable-DETR

**Figure 1:**
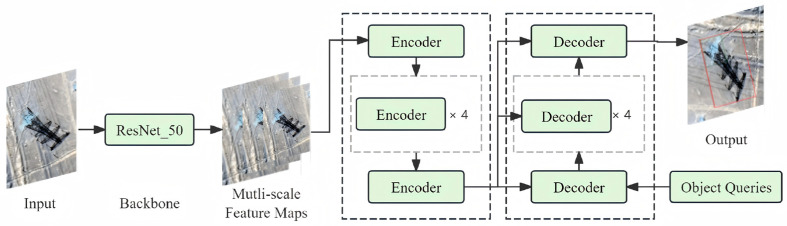
Overall architecture of the Deformable-DETR framework, including backbone, encoder, and decoder modules.

Deformable-DETR is adopted as the baseline detector in this work. Compared with traditional two-stage detectors, Deformable-DETR introduces multi-scale deformable attention, which significantly improves convergence speed and multi-scale representation capability, making it a strong baseline for remote sensing object detection tasks. The overall architecture is illustrated in Figure 1.

Specifically, the input image is first processed by a ResNet-50 backbone [36] to extract multi-scale feature maps. These feature maps are then flattened and combined with positional encodings to form feature sequences. The encoder takes these sequences as input and produces context-enhanced features through multi-scale deformable self-attention and feed-forward networks (FFNs).

The decoder receives K learnable object queries, which first interact via self-attention and then attend to encoder features through deformable cross-attention to extract target-related information, resulting in refined query representations. Finally, prediction heads generate classification scores and bounding box coordinates.

Object queries serve as carriers of target information, where each query corresponds to a potential object. During training, the Hungarian matching algorithm assigns queries to ground-truth objects in a one-to-one manner, enabling queries to learn semantic and positional information. However, in Deformable-DETR, query updates mainly rely on internal attention interactions within the decoder, lacking explicit guidance from global contextual information. Although encoder outputs contain rich spatial semantics, their influence on queries is indirect via cross-attention and lacks explicit channel-wise modulation. This limitation becomes more pronounced for small objects, whose features are more easily overwhelmed by complex backgrounds and thus require stronger global context guidance.

### 3.2. Refined Deformable-DETR

**Figure 2:**
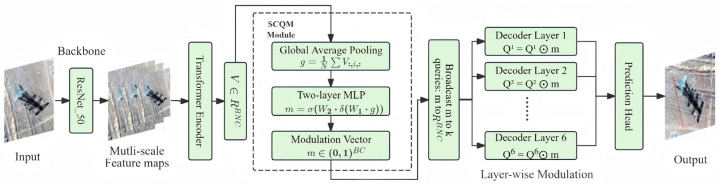
Overall architecture of the proposed Refined Deformable-DETR framework with the SCQM module.

To address the challenges of complex background interference and significant scale variation in electric pylon detection, we propose a Refined Deformable-DETR framework based on baseline model. The core idea is to enhance object query representations by incorporating global contextual information. The overall architecture is shown in Figure 2.

The proposed framework consists of three main components: a ResNet-50 backbone, a Transformer encoder, and a Transformer decoder. On top of this, a Spatial Context-aware Query Modulation (SCQM) module is introduced into the decoder to dynamically modulate object queries.

Specifically, the input image is first processed by the backbone to extract multi-scale features, which are then encoded by the Transformer encoder to obtain global context-aware representations. The encoder outputs are not only used as keys and values in cross-attention, but also serve as inputs to the SCQM module. Unlike the original Deformable-DETR, SCQM is applied before each decoder layer to recalibrate the current object query representations.

### 3.3. Spatial Context-Aware Query Modulation (SCQM)

**Figure 3:**
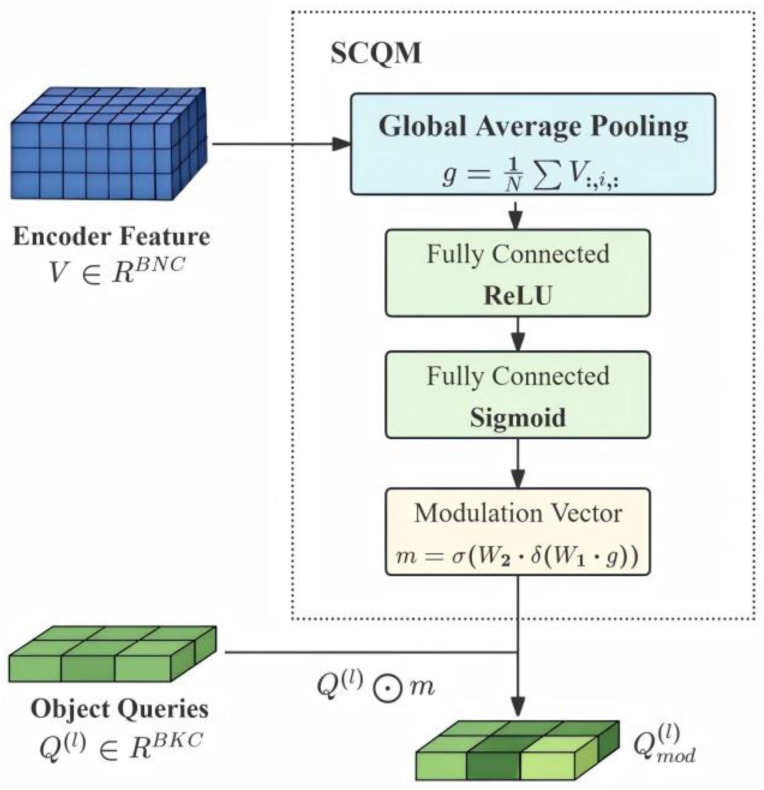
Overview of the proposed Spatial Context-aware Query Modulation (SCQM) module, including global context extraction, modulation vector generation, and query modulation.

To improve the representation ability of object queries in complex remote sensing scenarios, we propose the Spatial Context-aware Query Modulation (SCQM) module. Unlike feature recalibration methods such as SENet [31], which operate on feature maps, SCQM directly modulates object queries using global contextual information derived from the encoder. The overall structure is shown in Figure 3.

The SCQM module consists of three main steps: global context extraction, modulation vector generation, and query modulation.

Given an input image, features extracted by the backbone and encoder are denoted as $V\in R^{B\times N\times C}$, where *B* is the batch size, *N* is the number of spatial positions, and *C* is the channel dimension. The query representation at the *l*-th decoder layer is defined as $Q^{\left(l\right)}\in R^{B\times K\times C}$, where *K* is the number of object queries.

To obtain image-level contextual statistics, global average pooling (GAP) is applied along the spatial dimension of the encoder memory:

(1) $$g\;=\;\frac{1}{N}\;\sum_{i=1}^{N}V_{:,\;i\;,:}$$

Here, GAP compresses spatial responses into a channel-wise global descriptor $g\in R^{B\times C}$, rather than preserving precise object locations. Therefore, g is not used to directly localize pylons. Instead, it provides image-level contextual statistics for generating channel-wise query modulation weights, while the localization of pylons and shadows is still performed by the deformable cross-attention mechanism in the decoder through query-dependent sampling over multi-scale encoder features.

The global context vector is then passed through a two-layer multilayer perceptron (MLP) to generate a channel-wise modulation vector:

(2) $$m=\sigma (W_{2}\delta (W_{1}g))$$

where $W_{1}$ and $W_{2}$ are learnable weight matrices, δ(⋅) denotes the ReLU activation, and σ(⋅) denotes the sigmoid function. In our implementation, $W_{1}$ and $W_{2}$ are shared across all decoder layers. For each input image, a single shared modulation vector m is generated from the encoder memory and applied before each decoder layer. Therefore, *m* is written without a layer-specific superscript. In the general formulation, the intermediate dimension of the modulation MLP is controlled by the reduction ratio r, which provides a capacity–efficiency trade-off. Specifically, $W_{1}\in R^{C\times C/r}$ and $W_{2}\in R^{C/r\times C}$. In the final configuration, *r* = 1, meaning that the MLP preserves the full channel dimension without bottleneck compression. The resulting modulation vector is $m\in R^{B\times C}$, which reflects the channel-wise importance under the current image-level context. The influence of different r values is further evaluated in the ablation study.

The generated modulation vector is applied to object queries via channel-wise scaling:

(3) $$\;Q_{\mathrm{mod}}^{\left(l\right)}\;=\;Q^{\left(l\right)}\;⨀\;m$$

where ⨀ denotes element-wise (Hadamard) multiplication. The modulation vector m is broadcast along the query dimension. For each query $q_{k}^{\left(l\right)}\in R^{C}$, its *c*-th channel is scaled as:

(4) $$q_{k,c}^{\left(l\right)}\;=\;q_{k,c}^{\left(l\right)}\cdot m_{c}$$

In this formulation, each channel of each object query is adaptively amplified or suppressed according to the image-level contextual descriptor. Therefore, the calibration is performed in the channel dimension of query embeddings rather than in the spatial dimension of feature maps. This operation performs channel-wise recalibration on object queries, enabling them to adaptively incorporate global contextual information. SCQM does not replace or modify the deformable cross-attention mechanism. Instead, it recalibrates object queries before they enter each decoder layer. The subsequent deformable cross-attention still performs query-dependent spatial sampling on multi-scale encoder features, while the modulated queries provide an image-conditioned channel prior for feature interaction. Therefore, the global descriptor generated by GAP is used for query recalibration rather than direct spatial localization. Notably, SCQM does not increase the number of object queries, nor does it change feature dimensions or introduce additional supervision signals. Its computational overhead mainly comes from global pooling and two fully connected layers. Compared with the overall Deformable-DETR framework, SCQM introduces only a small number of additional parameters, while its actual inference-time overhead is quantitatively evaluated in Section 4.3.

### 3.4. Query Modulation Strength Analysis

In Transformer-based detectors, the modulation strength of object queries has a significant impact on detection performance. Previous studies have shown that adjusting the importance of queries or features via weighting mechanisms can alter model behavior. For example, DINO [10] introduces query-related modulation strategies, while QEDetr [37] employs a scaling factor to control query importance.

Inspired by these works, we introduce a modulation strength coefficient α to control the influence of global context on object queries. It should be noted that α is not a core component of the model, but a controllable variable used for systematic analysis.

Specifically, the modulation vector is adjusted via linear interpolation:

(5) $$\tilde{m}\;=\;1+\alpha (m-1)$$

where *α* ∈ [0, 1]. When *α* = 0, the modulation reduces to an identity mapping, making the model equivalent to the baseline. When *α* = 1, full modulation is applied. Accordingly, the query modulation is defined as:

(6) $${\tilde{Q}}_{mod}^{\left(l\right)}\;=\;Q^{\left(l\right)}\;\;⨀\;\;\tilde{m}$$

By varying *α*, we can analyze the relationship between modulation strength and detection performance, especially across different object scales. In this way, the contribution of global contextual information to query modulation can be systematically varied, and the corresponding ablation results are reported in Section 4.4.3.

## 4. Results and Analysis

### 4.1. Experimental Environment

All experiments were conducted on a server equipped with four NVIDIA A100-SXM4-80GB GPUs (NVIDIA, Santa Clara, CA, USA) and an Intel Xeon Gold 6326 CPU (Intel, Santa Clara, CA, USA). The software environment includes CUDA 11.8 and the PyTorch 2.1.2 deep learning framework. All models, including the baseline Deformable-DETR and the proposed method, were implemented based on the MMDetection 3.3.0 toolbox.

The models were trained using the AdamW optimizer with an initial learning rate of 2 × 10^−4^ and a weight decay of 1 × 10^−4^. A multi-step learning rate schedule was adopted, where the learning rate was reduced by a factor of 0.1 at the 40th epoch. The total number of training epochs was set to 50, with a batch size of 8. Gradient clipping was applied to stabilize training, with the maximum gradient norm set to 0.1.

For data augmentation, random horizontal flipping, multi-scale training, and random cropping were employed. During inference, the input images were processed by the testing pipeline and resized to 800 × 800 for model evaluation. The key hyperparameter settings are summarized in Table 1.

### 4.2. Datasets and Evaluation Metrics

#### 4.2.1. EPRD

To evaluate the proposed method, experiments were first conducted on a self-constructed Electric Pylon Remote Sensing Dataset (EPRD), which was developed by our research team. The dataset consists of 1340 high-resolution satellite images with a spatial resolution of 0.6 m, and all images are resized to 512 × 512 pixels. A total of 2178 electric pylon instances is annotated, where each bounding box covers both the pylon structure and its shadow, enabling joint modeling of structural and contextual information [3]. All annotations follow the Common Objects in Context (COCO) format [38].

**Figure 4:**
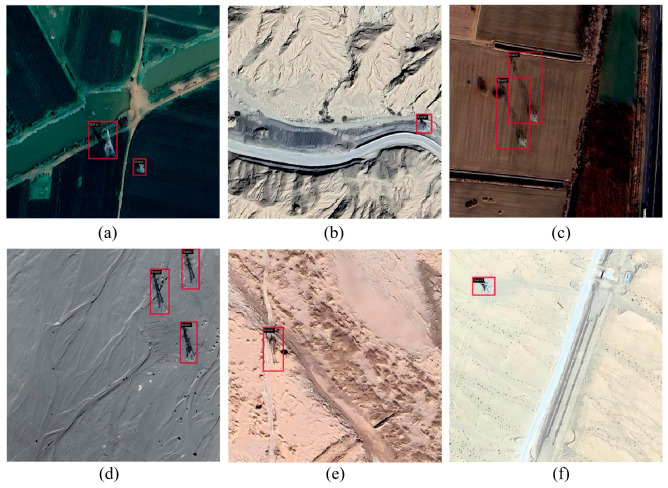
Representative samples of the EPRD. (**a**,**c**) Farmland scenes with linear background structures. (**b**,**e**) Mountainous or hilly scenes with complex terrain textures. (**d**,**f**) Bare-land and desert-road scenes with low-contrast backgrounds and shadow variations. The red boxes denote annotated electric pylons together with their associated shadows, illustrating variations in object scale, shadow extent, and background complexity.

The high-resolution optical imagery in EPRD is collected from multiple sources, including Google Earth, Jilin-1 satellite imagery, Century Spatial, and SuperView satellite imagery. The dataset covers diverse geographic environments such as mountainous regions, farmlands, and desert areas, ensuring significant variability in background complexity and object appearance. This diversity enhances the robustness and generalization capability of the proposed model. Figure 4 presents several representative samples from the EPRD, illustrating variations in object scale, background complexity.

The dataset is split into training, validation, and test sets with a ratio of approximately 6:2:2, including 741 (train), 242 (val) and 357 (test) images, with 1210, 407 and 561 annotated instances, respectively. To analyze performance across different object scales, targets are categorized based on bounding-box area into small (<32 × 32 pixels), medium (32 × 32 to 96 × 96 pixels), and large (>96 × 96 pixels) objects, following the COCO area-based definition. Here, object scale refers to the pixel area of annotated bounding boxes rather than the physical height or real-world size of pylons. The distribution of object scales is summarized in Table 2.

As shown in Table 2, the EPRD exhibits significant scale variation, with medium-sized objects dominating the dataset while small objects still account for a non-negligible proportion. This distribution poses challenges for multi-scale representation learning and provides a suitable benchmark for evaluating detection performance in complex remote sensing scenarios.

#### 4.2.2. EPD

To further evaluate the generalization capability of the proposed method, experiments were conducted on the public Electric Pylon Dataset (EPD) [39]. This dataset consists of approximately 1500 high-resolution optical remote sensing images with a spatial resolution of about 1 m and a unified size of 1024 × 1024 pixels.

The dataset contains over 3000 annotated pylon instances under diverse and complex backgrounds, including varying terrain types and imaging conditions. To maintain consistency with the EPRD and experimental settings, the EPD was re-split into training, validation, and test sets with a ratio of 6:2:2.

All experiments on EPD were conducted using the same training strategy and evaluation metrics as those used for EPRD, ensuring fair comparison and enabling reliable assessment of model generalization across different data distributions.

#### 4.2.3. Evaluation Metrics

Following the COCO evaluation protocol [38], which is a widely used benchmark protocol for object detection and instance segmentation, the Average Precision (AP) is adopted as the primary evaluation metric. AP is computed as the mean precision over multiple Intersection over Union (IoU) thresholds ranging from 0.50 to 0.95 with a step size of 0.05, providing a comprehensive assessment of both classification and localization performance.

In addition, several auxiliary metrics are reported, including AP_50_ (IoU = 0.50), AP_75_ (IoU = 0.75), and AP for different object scales, namely AP_S_ (small), AP_M_ (medium), and AP_L_ (large). AP_50_ reflects the general detection capability, while AP_75_ emphasizes localization accuracy. The scale-specific metrics (APs, AP_M_, AP_L_) are used to evaluate detection performance across different object scales defined by bounding-box area.

### 4.3. Main Results on EPRD

To evaluate the effectiveness of the proposed SCQM module, we compare it with several representative Transformer-based detectors under the same experimental settings, including DETR, Conditional DETR, DAB-DETR, and the baseline Deformable-DETR. All models are trained under identical conditions as described in Section 4.1 to ensure fair comparison. The quantitative results are presented in Table 3.

As shown in Table 3, the proposed method achieves the best overall performance. Specifically, the AP improves from 72.7% to 74.1% compared to the baseline Deformable-DETR, yielding a gain of 1.4%. This improvement indicates that incorporating SCQM enables the model to better exploit global contextual information, thereby enhancing detection accuracy.

Notably, the proposed method demonstrates a more significant improvement on small objects. The AP_S_ increases from 47.2% to 50.9%, with a gain of 3.7%, suggesting that the proposed query modulation mechanism effectively enhances the representation capability for small-scale targets. This is particularly beneficial in remote sensing scenarios, where small objects are often difficult to distinguish from complex backgrounds.

In addition, improvements are also observed in localization accuracy. The AP_75_ increases from 87.6% to 89.1%, indicating that the modulated queries can produce more precise bounding box predictions. Meanwhile, consistent gains are achieved in AP_M_ and AP_L_, demonstrating that the proposed method is not limited to small objects but generalizes well across different scales.

To further evaluate efficiency, we compare the baseline Deformable-DETR and the proposed Refined Deformable-DETR in terms of parameters, floating-point operations (FLOPs), and inference speed in frames per second (FPS). FLOPs were calculated under the same testing pipeline with an input size of 800 × 800. FPS was measured on the entire EPRD test set with batch size 1 after 20 warm-up iterations on a single NVIDIA A100 GPU. The results are shown in Table 4.

**Figure 5:**
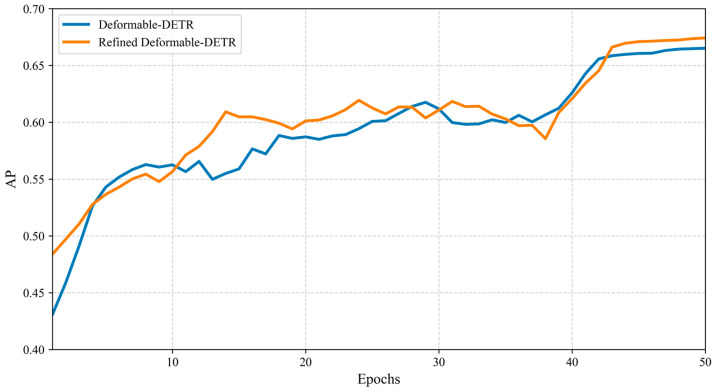
Training convergence curves of the baseline Deformable-DETR and the proposed method on the validation set.

As shown in Table 4, Refined Deformable-DETR has comparable computational complexity to the baseline Deformable-DETR. Specifically, the number of parameters increases slightly from 40.10 M to 40.23 M, corresponding to only 0.13 M additional parameters. The reported FLOPs remain the same at 123.0 G under the adopted measurement precision. In terms of inference speed, FPS decreases from 27.0 to 24.9 after introducing SCQM. Nevertheless, the proposed method improves AP from 72.7% to 74.1% and AP_S_ from 47.2% to 50.9%. These results indicate that SCQM brings a clear accuracy gain, especially for small objects, while introducing only a limited computational overhead. To further analyze the training behavior, the convergence curves of Deformable-DETR and the proposed method on the validation set are shown in Figure 5. It can be observed that the proposed method not only achieves higher final accuracy but also exhibits faster convergence and more stable training dynamics, further validating the effectiveness of query modulation.

**Figure 6:**
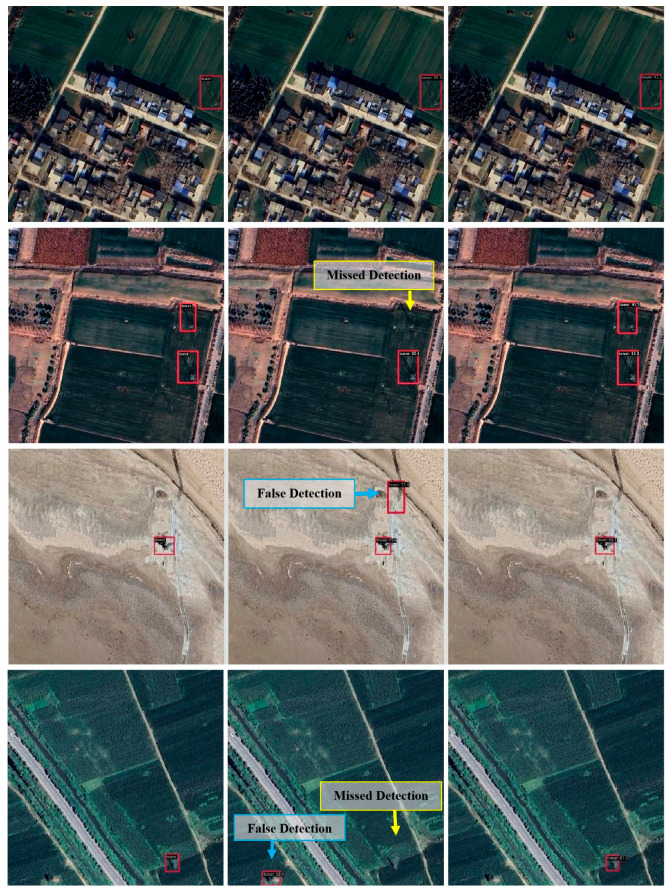

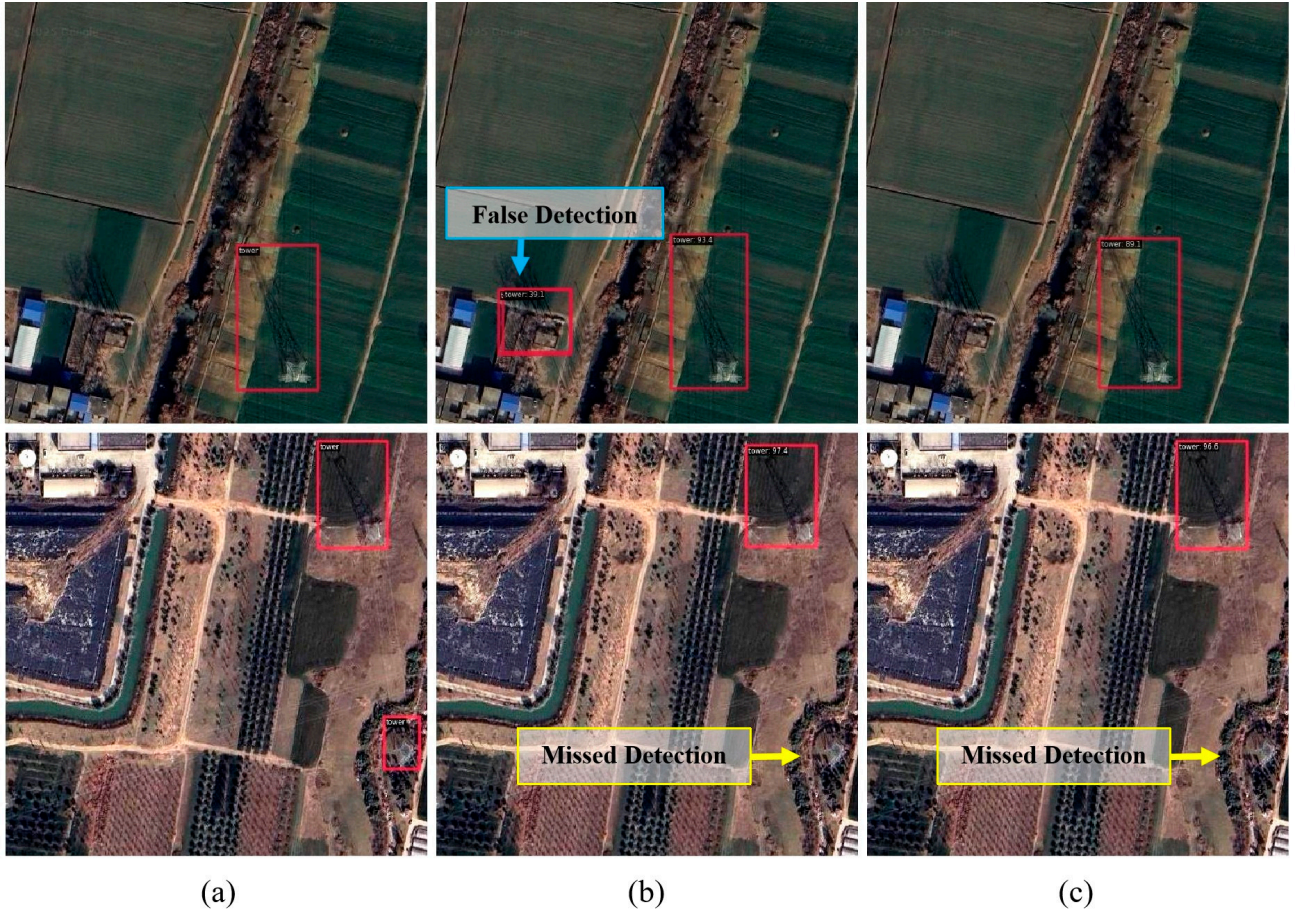
Qualitative comparison of detection results on representative EPRD test images. (**a**) Ground-truth. (**b**) Detection results of the baseline Deformable-DETR. (**c**) Detection results of the proposed Refined Deformable-DETR. The proposed method produces fewer missed detections and false positives in complex backgrounds and provides more accurate localization for pylon-shadow structures.

To provide a more intuitive comparison between the proposed method and the baseline model in complex remote sensing scenarios, several representative detection results on the test set are visualized, as shown in Figure 6. It can be observed that, due to the high visual similarity between electric pylons and their surrounding backgrounds, the baseline Deformable-DETR suffers from noticeable false positives and missed detections. In contrast, the proposed method with the SCQM module achieves more accurate detection results, with fewer false detections and missed targets, as well as more precise bounding box localization. These qualitative results further demonstrate that incorporating spatial context-aware query modulation enhances the model’s ability to distinguish target objects from complex backgrounds, thereby improving detection robustness in challenging remote sensing environments.

**Figure 7:**
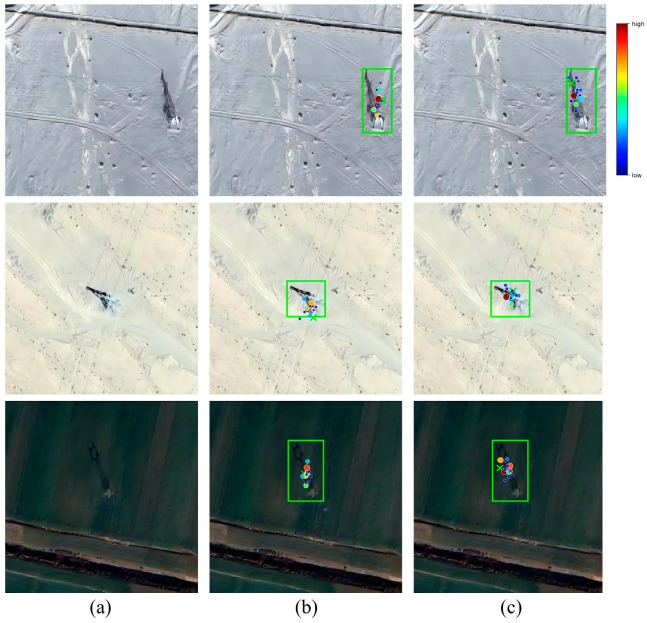
Matched-query deformable cross-attention visualization. (**a**) Cropped input images. (**b**) Sampling locations and attention weights of matched object queries from the baseline Deformable-DETR. (**c**) Sampling locations and attention weights of matched object queries from the proposed Refined Deformable-DETR. Green boxes denote the selected reference predicted bounding boxes. Point color and size indicate normalized attention weights, where warmer colors and larger markers correspond to higher responses.

However, bounding-box visualization only reflects the final detection results and cannot directly reveal how object queries interact with image features during decoding. Motivated by prior studies on deformable attention and object-query behavior in DETR-based detectors [40,41], we further visualize the last-layer deformable cross-attention sampling points and their corresponding attention weights for matched object queries, as shown in Figure 7. Since Deformable-DETR adopts sparse deformable cross-attention rather than dense global attention, this visualization represents a limited set of sampling locations instead of dense attention heatmaps. The matched queries are selected according to bounding-box overlap or center distance, and the associated sampling locations and attention weights are mapped back to the original image space for visualization. Therefore, it is used as a supplementary qualitative observation rather than as direct proof of attention redistribution.

As shown in Figure 7, both the baseline Deformable-DETR and the proposed Refined Deformable-DETR can generate sampling responses around the selected pylon targets. The visual differences between the two models are relatively subtle, which is expected due to the sparse sampling nature of deformable cross-attention. In the displayed examples, several sampling responses of the proposed method appear around the pylon body, shadow, and adjacent contextual regions. This observation is consistent with the quantitative improvements reported in Table 3 and provides a qualitative reference for understanding the matched-query sampling behavior.

It should be noted that SCQM does not directly impose spatial constraints on sampling locations or attention weights. Instead, it recalibrates object queries before deformable cross-attention through an image-conditioned channel prior derived from the encoder memory. Therefore, Figure 7 is intended to provide a supplementary qualitative view of matched-query sampling behavior, while the effectiveness of SCQM is mainly supported by quantitative comparisons and ablation studies.

### 4.4. Ablation Studies

#### 4.4.1. Effect of the Reduction Ratio r

To evaluate the influence of the reduction ratio r, we compare different intermediate dimensions in the SCQM MLP. The reduction ratio controls the capacity–efficiency trade-off of the modulation MLP: a smaller r preserves a larger hidden dimension and stronger modulation capacity, whereas a larger r introduces stronger bottleneck compression and reduces the number of parameters. The results are shown in Table 5.

As shown in Table 5, increasing r slightly reduces the number of parameters, from 40.23 M at r = 1 to 40.11 M at r = 16. However, this parameter reduction does not lead to better detection performance. The model achieves the best overall AP of 74.1% and the best APS of 50.9% when r = 1. When r is increased to 4 and 8, the overall AP slightly decreases to 73.9%, while APS decreases to 47.4% and 49.1%, respectively. When a stronger bottleneck is adopted with r = 16, the performance further drops to 73.2% AP and 42.5% APs.

These results indicate that excessive bottleneck compression may weaken the modulation capacity of SCQM, especially for small objects. Since GAP already compresses the spatial dimension into an image-level descriptor, further reducing the channel dimension may discard useful channel interactions for generating effective query modulation weights. Therefore, r = 1 is adopted in the final configuration of Refined Deformable-DETR.

#### 4.4.2. Effect of Pooling Strategy

To evaluate whether the GAP-based context descriptor is suitable for SCQM, we compare different pooling strategies for generating the global context vector, including global average pooling (GAP), global max pooling (GMP), and their additive combination (GAP+GMP). For a fair comparison, all other settings are kept unchanged, including the reduction ratio r = 1, applying SCQM before each decoder layer, and the same training strategy. The results are shown in Table 6.

As shown in Table 6, GMP achieves the highest overall AP of 74.5%, which is 0.4% higher than that of GAP. However, its AP_S_ decreases substantially from 50.9% to 46.8%. This indicates that emphasizing only the strongest spatial responses may benefit some medium-sized instances but is less stable for small pylons. The additive combination of GAP and GMP does not further improve performance, yielding 73.5% AP and 45.1% AP_S_. In contrast, GAP achieves the best small-object performance, with AP_S_ reaching 50.9%. Since SCQM uses the global descriptor to generate image-level channel-wise query modulation weights rather than to directly localize objects, GAP-based averaged contextual statistics are more suitable for providing stable query recalibration. Therefore, GAP is adopted in the final SCQM configuration.

#### 4.4.3. Effect of Query Modulation Strength

To further analyze the effect of the modulation strength coefficient *α* introduced in Section 3.4, a series of ablation experiments are conducted under different values of *α*. By adjusting *α*, the influence of global contextual information on object queries can be controlled, allowing us to investigate the relationship between modulation strength and detection performance. The results are summarized in Table 7.

When *α* = 0, the modulation vector degenerates to an identity vector, and the model is equivalent to the baseline Deformable-DETR. When *α* > 0, image-conditioned channel-wise modulation is introduced into the object queries. Therefore, varying *α* allows us to analyze how different degrees of query recalibration affect detection performance.

As shown in Table 7, introducing a small modulation strength already brings a clear improvement in overall AP. Specifically, AP increases from 72.7% at *α* = 0 to 73.8% at *α* = 0.25. This suggests that even weak image-conditioned query modulation can effectively recalibrate the original object queries. However, the performance gain is not linearly proportional to *α*. When *α* increases from 0.25 to 1, the overall AP changes within a relatively narrow range, while APS increases from 47.3% to 50.9%.

This result indicates that SCQM may also play a regularization-like role in query updating by introducing image-conditioned channel priors, especially under weak modulation strengths. Meanwhile, the full modulation setting *α* = 1 achieves the best overall AP and the best small-object AP in our experiments. Therefore, *α* = 1 is adopted as the final configuration. Overall, these results suggest that moderate query recalibration is already effective, while the full SCQM setting provides the most favorable balance between overall detection performance and small-object detection capability.

### 4.5. Generalization Results on the EPD

The generalization performance of the proposed method is further evaluated on the EPD, with results presented in Table 8. Compared with other DETR-based methods, the proposed Refined Deformable-DETR achieves the best performance on this dataset. Specifically, the overall AP improves from 49.6% to 50.8% compared to the baseline Deformable-DETR, yielding a gain of 1.2%. This improvement indicates that the proposed method maintains stable performance gains under cross-dataset scenarios, demonstrating its generalization capability. It is worth noting that, in the EPD, all annotated objects have bounding box areas smaller than 96 × 96 pixels. Therefore, the AP_L_ metric is not applicable and is not reported in this experiment.

## 5. Discussion

This work investigates the optimization of object query representations in Transformer-based detectors and proposes the SCQM module based on the hypothesis that incorporating global contextual information can improve detection performance in complex remote sensing scenarios. The experimental results validate that SCQM consistently enhances both overall detection performance and small-object detection capability.

From an application perspective, automatic localization of electric pylons from optical satellite imagery can support large-area power infrastructure inspection, infrastructure inventory updating, post-disaster damage assessment, and maintenance planning. Compared with manual inspection or UAV-based surveys, satellite-based detection provides broader spatial coverage and is more suitable for preliminary screening over large regions. The present work focuses on algorithmic development and dataset-level validation rather than a complete industrial deployment system, but the proposed framework has practical potential for remote sensing-based power infrastructure monitoring.

Unlike existing methods that primarily focus on feature enhancement or query initialization, the proposed approach explicitly models query representations by introducing global semantic information from the encoder into the decoder. This channel-wise modulation mechanism enables object queries to adapt to image-specific context, which is particularly beneficial for small objects that heavily rely on contextual cues in remote sensing imagery.

The ablation study further indicates that the effect of modulation strength is nonlinear, suggesting that SCQM may provide both image-conditioned query recalibration and a regularization-like effect on query updating.

Moreover, the consistent performance gains observed on the EPD demonstrate that the proposed method generalizes well across different data distributions. This suggests that SCQM captures a transferable context modulation mechanism rather than relying on dataset-specific characteristics.

Despite these advantages, several limitations remain. First, although the EPRD contains diverse backgrounds and different pylon-shadow appearances, it does not include reliable seasonal or meteorological metadata. Therefore, strict winter/summer or cloud-covered subgroup experiments cannot be conducted in the current study. Since shadow length and contrast may vary with season, illumination angle, and cloud conditions, the robustness of the proposed method under different seasonal and atmospheric conditions should be further evaluated in future work by collecting multi-season and cloud-affected satellite images. Second, the use of global average pooling may limit the modeling of fine-grained spatial structures, and the current design only performs channel-wise modulation without considering spatial or inter-query interactions. Future work will explore more refined context modeling strategies and richer query modulation mechanisms to further improve detection performance.

## 6. Conclusions

In this paper, a Refined Deformable-DETR framework is proposed to address the challenges of background interference and scale variation in electric pylon detection from remote sensing images. The proposed SCQM module introduces global context-aware channel-wise modulation to enhance object query representations in the Transformer decoder.

Experimental results on the self-constructed EPRD demonstrate that the proposed method achieves improved detection performance compared with the baseline Deformable-DETR, with particularly notable gains in small-object detection. Additional evaluation on the public EPD further confirms the generalization capability of the proposed method across different data distributions. Moreover, the efficiency analysis and ablation studies further support the effectiveness and practicality of the SCQM design, while the deformable cross-attention visualization provides supplementary qualitative observations of matched-query behavior.

Despite these improvements, several issues remain for future investigation. The current SCQM design relies on global average pooling and performs only channel-wise query modulation, which may limit its ability to model fine-grained spatial-context and inter-query relationships. Future work will explore more refined spatial-context modeling strategies, richer query modulation mechanisms, and multi-season or cloud-affected satellite imagery to further improve the robustness of electric pylon detection in complex remote sensing scenarios.

## Figures and Tables

**Table 1:** Hyperparameter configuration.

Hyperparameter	Setting
Backbone	ResNet-50
Optimizer	AdamW
Number of object queries	300
Epochs	50
Train Batch Size	8
Initial Learning Rate	2 × 10^−4^
Weight decay	1 × 10^−4^
SCQM reduction ratio r	1

**Table 2:** Distribution of object scales in EPRD.

EPRD	Images	Total Objects	Small	Medium	Large
Train	741	1210	5.9%	78.0%	16.1%
Validation	242	407	11.3%	81.8%	6.9%
Test	357	561	6.3%	82.5%	11.2%
Total	1340	2178	7.0%	79.9%	13.1%

**Table 3:** Quantitative comparison of different methods on the EPRD.

Method	Epochs	AP	AP_50_	AP_75_	AP_S_	AP_M_	AP_L_
DETR	150	73.3	96.1	87.5	37.2	73.0	86.2
DAB-DETR	50	70.9	95.3	85.2	40.8	70.7	80.1
Conditional-DETR	50	71.4	96.2	87.2	35.3	70.7	84.5
Deformable-DETR	50	72.7	96.1	87.6	47.2	72.5	82.4
Refined Deformable-DETR	50	74.1	96.2	89.1	50.9	73.7	85.0

**Table 4:** Computational complexity and inference efficiency comparison on the EPRD test set.

Method	Epochs	Params (M)	FLOPs (G)	FPS	AP	AP_S_
Deformable-DETR	50	40.10	123.0	27.0	72.7	47.2
Refined Deformable-DETR	50	40.23	123.0	24.9	74.1	50.9

**Table 5:** Ablation study on the reduction ratio r in SCQM.

r	Hidden dim.	Params (M)	AP	AP_50_	AP_75_	AP_S_	AP_M_	AP_L_
1 (SCQM)	256	40.23	74.1	96.2	89.1	50.9	73.7	85.0
4	64	40.13	73.9	96.4	89.4	47.4	73.8	84.2
8	32	40.12	73.9	96.6	87.0	49.1	73.6	85.9
16	16	40.11	73.2	95.5	87.9	42.5	72.6	86.5

**Table 6:** Ablation study on different pooling strategies in SCQM.

Pooling Strategy	Params (M)	AP	AP_50_	AP_75_	AP_S_	AP_M_	AP_L_
GAP (SCQM)	40.23	74.1	96.2	89.1	50.9	73.7	85.0
GMP	40.23	74.5	96.4	89.3	46.8	74.4	83.8
GAP+GMP	40.23	73.5	95.5	87.6	45.1	73.5	83.9

**Table 7:** Ablation results of different query modulation strengths *α* on the EPRD.

*α*	AP	AP_50_	AP_75_	AP_S_	AP_M_	AP_L_
0	72.7	96.1	87.6	47.2	72.5	82.4
0.25	73.8	97.1	88.6	47.3	73.7	83.1
0.5	73.7	95.8	90.1	47.9	73.6	84.2
0.75	73.4	95.9	90.1	49.0	73.2	84.6
1 (SCQM)	74.1	96.2	89.1	50.9	73.7	85.0

**Table 8:** Performance comparison of different methods on the EPD.

Method	Epochs	AP	AP_50_	AP_75_	AP_S_	AP_M_	AP_L_
DETR	150	43.6	81.3	39.5	36.0	64.0	-
DAB-DETR	50	45.5	84.1	44.3	40.3	67.9	-
Conditional-DETR	50	46.6	87.3	44.4	42.1	68.8	-
Deformable-DETR	50	49.6	88.4	50.4	45.0	68.2	-
Refined Deformable-DETR	50	50.8	90.2	54.1	46.9	70.1	-

## Data Availability

The EPD can be downloaded from the following website: https://universe.roboflow.com/robin-public/electric-pylon-detection-in-rsi, accessed on 19 November 2025.
